# Fast and Efficient XML Data Access for Next-Generation Mass Spectrometry

**DOI:** 10.1371/journal.pone.0125108

**Published:** 2015-04-30

**Authors:** Hannes L. Röst, Uwe Schmitt, Ruedi Aebersold, Lars Malmström

**Affiliations:** 1 Department of Biology, Institute of Molecular Systems Biology, ETH Zurich, CH-8093 Zurich, Switzerland; 2 Ph.D. Program in Systems Biology, University of Zurich and ETH Zurich, CH-8057 Zurich, Switzerland; 3 ID Scientific IT Services, ETH Zurich, CH-8092 Zurich, Switzerland; 4 Competence Center for Systems Physiology and Metabolic Diseases, CH-8093 Zurich, Switzerland; 5 Faculty of Science, University of Zurich, CH-8057 Zurich, Switzerland; 6 S3IT, University of Zurich, CH-8057 Zurich, Switzerland; UGent / VIB, BELGIUM

## Abstract

**Motivation:**

In mass spectrometry-based proteomics, XML formats such as mzML and mzXML provide an open and standardized way to store and exchange the raw data (spectra and chromatograms) of mass spectrometric experiments. These file formats are being used by a multitude of open-source and cross-platform tools which allow the proteomics community to access algorithms in a vendor-independent fashion and perform transparent and reproducible data analysis. Recent improvements in mass spectrometry instrumentation have increased the data size produced in a single LC-MS/MS measurement and put substantial strain on open-source tools, particularly those that are not equipped to deal with XML data files that reach dozens of gigabytes in size.

**Results:**

Here we present a fast and versatile parsing library for mass spectrometric XML formats available in C++ and Python, based on the mature OpenMS software framework. Our library implements an API for obtaining spectra and chromatograms under memory constraints using random access or sequential access functions, allowing users to process datasets that are much larger than system memory. For fast access to the raw data structures, small XML files can also be completely loaded into memory. In addition, we have improved the parsing speed of the core mzML module by over 4-fold (compared to OpenMS 1.11), making our library suitable for a wide variety of algorithms that need fast access to dozens of gigabytes of raw mass spectrometric data.

**Availability:**

Our C++ and Python implementations are available for the Linux, Mac, and Windows operating systems. All proposed modifications to the OpenMS code have been merged into the OpenMS mainline codebase and are available to the community at https://github.com/OpenMS/OpenMS.

## Introduction

In current mass spectrometry (MS) based proteomics, a mass spectrometer is used to sequentially record mass spectra consisting of mass to charge (*m/z*) *vs*. intensity measurements [1]. Most commonly, the mass spectrometer is connected on-line to a liquid chromatography (LC) system that separates analytes contained in a complex sample. Next, the analytes eluting at any given time from the column are ionized and measured by the mass spectrometer. The acquired spectra from one LC-MS run are then stored in a single file on disk, usually associated with additional meta-information like chromatographic retention time or the appropriate MS-level and precursor selection range (for tandem MS) [2, 3]. While instrument vendor software generally only supports the vendor’s respective proprietary storage format, several open formats for storing mass spectrometric data have emerged over the last decade. Most notable among these are the mzData, mzXML and mzML data formats, mzML being the standard format officially supported by the PSI (Proteome Standard Initiative) [4–6]. The availability of open data formats has led to the development of open-source algorithms performing a multitude of tasks in computational proteomics, ranging from database identification and subsequent statistical scoring of peptide spectrum matches [7–9] to other tasks such as MS1 feature identification [10–12] or even targeted data analysis [13, 14]. The field of proteomics has profited greatly from these standardized data exchange formats through the availability of instrument vendor independent open-source software, a cornerstone of reproducible and transparent data analysis.

Even though these formats have enabled numerous advances in computational proteomics, the high complexity of XML (extensible markup language) also posed challenges to writers of proteomics software. Many published tools feature their own XML parsing implementation and each tool often only supports a subset of the standardized features. Very few tools achieve full standard compliance or even support for multiple standard formats. Some of the more popular tools that strive towards standard-compliant implementation of (one or more) PSI formats are the ProteoWizard library [15, 16], mspire [17], jmzML [18] mzR [15], the PRIDE toolsuite [19], jmzReader [20] and the OpenMS library [12] (see also Perez-Riverol et al. [21] for a recent discussion of different open source software libraries in mass spectrometry). Specifically, the OpenMS mass spectrometric software environment consists of a set of flexible algorithms, tools and visualization solutions that can be accessed at the level of a shared C++ library, the level of interactive Python bindings and the level of workflows, where graphical workflow managers allow the creation of complete proteomics data analysis pipelines [12, 22]. OpenMS supports multiple file formats standardized by the PSI and in addition to its core C++ library, it provides over 150 associated stand-alone executable tools which can be arranged in a set of adaptable workflows [11]. The OpenMS architecture, which uses a shared library for core functionality, makes each improvement in the core library immediately accessible to all associated stand-alone tools. The library itself is written in C++ with a recent addition of complete API-level bindings for the Python language [23], thus addressing a large swath of proteomics developers, ranging from C++ specialists to versatile Python developers.

Recent advancements in MS instrumentation have increased data quality but also led to an increase in data volume. Specifically, next-generation mass spectrometric technologies such as SWATH-MS produce datasets on high resolution TOF instruments which can routinely exceed 50 GB in file size when converted to mzML and challenge existing software architectures [24]. As a consequence, the efficient parsing and representation of data structures in memory have become critical for mass spectrometric software libraries.

The OpenMS data model, for example, loads mass spectrometric raw data completely into system memory and will therefore not work efficiently when presented with a file that exceeds the memory capabilities of the system. In general, this means that the memory requirements are equal or larger than the disk size of a data file, e.g. a 50 GB data file would require at least 50 GB of system memory to process. This limitation hindered data analysis with OpenMS dramatically and proposed remedies, such as splitting XML files into multiple smaller files to process them separately [14], do not present feasible long-term solutions. To truly make OpenMS compatible with next-generation MS technology, a new software architecture was warranted to support multiple orthogonal data access techniques for different use-cases. We reasoned that an architectural re-design within OpenMS would be highly beneficial for the proteomics community since a large number of tightly integrated C++ tools (as well as Python scripts using pyOpenMS) would have direct access to such an improved architecture.

Here we present a high-performance, low-memory API (application programming interface) to access raw mass spectrometric data implemented in C++ and Python, two common programming languages in computational proteomics. The features of our software library include support for indexed mzML, support for low-memory random access as well as event-driven sequential access to spectra and chromatograms and increased speed in parsing XML-based proteomics data files. The proposed API is available to the over 150 proteomic tools utilizing the OpenMS C++ framework and to all Python tools utilizing the pyOpenMS framework, therefore providing an improvement in a core area of the library which will impact a multitude of proteomics analysis tools and thus proteomic processes and studies.

## Materials and Methods

### Code modifications

We modified the existing OpenMS C++ API to include a set of functions which allow efficient processing of large XML datastructures with minimal memory expenditure. Specifically, we added the IndexedMzMLFileLoader class to the API which allows loading of an indexed mzML file and returns an object of type OnDiscMSExperiment to the client. This provides random read access (“lazy loading”) to any spectrum or chromatogram present in the indexed mzML file by loading only the currently requested data structure into memory. In addition, we added the CachedmzML class to the API which is able to read a cached mzML file from disk. For event-driven data processing, we implemented transform functions (inspired by the std::transform concept in the C++ standard template library) for the MzMLHandler and the MzXMLHandler objects which apply a requested transformation to a given file on disk without ever fully loading the file into memory. The user can provide the transformation operation through a class derived from Interfaces::IMSDataConsumer (or in Python, a class that implements all four required functions, see online documentation and S1 Text).

Several implementations of the Interfaces::IMSDataConsumer are provided and implement basic functions such as writing to the mzML format, writing to a cached mzML format, applying a given function pointer to all data, chaining consumers together to perform multiple operations in a pre-defined order or simply perform no operation (to be used at a place where a Interfaces::IMSDataConsumer object is expected but no operation is desired).

### Code analysis

By dynamic analysis of the XML-parsing code of OpenMS using the “callgrind” profiling tool, we were able to identify critical performance bottlenecks. Specifically, we identified performance issues during conversion of Base64 encoded data strings (as found in the mzML and mzXML data formats) to floating point values as well as during the construction of MSSpectrum instances. We were able to improve the performance through optimized Base64 parsing and through parallelization of the critical code sections, achieving an overall performance boost of a factor of 4 or more compared to the default OpenMS 1.11 code.

### Performance measurement

All measurements of speed and system memory were performed on the same Red Hat Enterprise Linux server using a custom built version of OpenMS which incorporated all the described patches and reporting code for speed and memory requirements. A small sample application, TICCalculator, was written to compare the speed and memory requirements of the different algorithms described in the text. To evaluate single-threaded parsing, the -threads command line parameter was set to 1 while it was set to 16 for the multi-threaded comparisons. To measure the parsing speed of the ProteoWizard library, a sample program was built against the ProteoWizard software library (revision 7261, committed 2015-03-06); all related code can be found at https://github.com/hroest/pwiz_readspeed. Resident size was used for all memory measurements. All measurements were performed three times (after two burn-in runs) and averages are reported. As a test file, a single file from Röst et al. [14] was chosen and test files of different sizes were generated using the OpenMS FileFilter utility by selecting a retention time range of 100, 250, 500 and 750 seconds from the original input file, starting 2000 seconds into the original file. The comparison of all algorithms was only performed on the small, filtered files since the memory inefficient algorithms could not handle the full file (60 GB size). We decided against reporting CPU time and in favor of walltime since walltime is closer to the user-experienced wait time which is relevant here. The source code for the TICCalculator sample application can be downloaded from https://github.com/hroest/OpenMS/tree/feature/measureMemoryConsumption. All comparisons shown in the main text refer to the C++ API, see S1 Text for performance benchmarks of the Python API.

### Availability

All described is currently available through https://github.com/OpenMS/OpenMS and has been merged into the main OpenMS development branch. Documentation for the different file access modes by read and write use-case are provided through the OpenMS documentation including three fully functional and nightly tested sample programs (see documentation at http://ftp.mi.fu-berlin.de/OpenMS/documentation/html/tutorial_format.html).

## Results

Here we present a fast and versatile C++ and Python library for reading and writing XML data formats used in MS-based proteomics which is based on the OpenMS framework. The library provides raw data access for different usage scenarios by allowing users to select from several access modes tailored for a specific use-case (see Table 1). One access mode is optimized for sequential data processing (“event-driven”), one for frequent random access (“cache”), one for infrequent random access (“indexed”), while the original implementation suited for small-scale data (“in-memory”) is also still available. Specifically, the new features described here and implemented in OpenMS include:

**Table 1:** Available access types of raw mass spectrometric data through OpenMS. This paper describes the implementation and use of the “random access on disk” (indexed mzML and cached mzML) and the “event-driven” access types and compares it to the previously available “random access in memory” method.

**Access Type**		**Use case**
Random Access in Memory	Read	Fast access to small XML datastructures
	Write	Fast storage of small XML datastructures
Random Access on Disc (indexed)	Read	Access for infrequent random access
	Write	Only sequential writing possible (see below)
Random Access on Disc (cached)	Read	Ultra-fast access for frequent random access
	Write	Only sequential writing possible (see below)
Event-driven processing	Read	Sequential read access suitable for compressed or non-indexed files
	Write	Sequential write interface for mzML, indexed mzML, cached mzML

### Random read access to disk

A new class has been added to the API which provides read access to spectra present on the hard disk using a pre-computed index. The addition of the index at the end of the XML file is implemented according to the extended mzML_idx standard (mzML1.1.1_idx.xsd), which is now fully supported by the modified OpenMS library using our extensions. The client C++ or Python code is able to get access to the total number of spectra and chromatograms and retrieve individual data points directly by their identifier. This allows random access to mass spectrometric data in files of virtually any size.

For certain applications, performing random seeks inside an XML file on disk may be prohibitively expensive since for each access, the underlying XML data format will have to be parsed and decoded. This may lead the algorithm to parse the same XML data multiple times if access requests cannot be bundled together, resulting in performance bottlenecks. To address this specific problem, we next implemented a simple binary representation of mzML inside OpenMS which provides random access to mass spectrometric data as well as all meta-data on request. The implemented API encapsulates the binary representation and provides convenient access to raw data and meta-data in a transparent fashion such that the client application does not need to have knowledge about the actual representation of the data. In practice, the user would first cache the mzML file to produce a .cachedMzML file which contains all spectral and chromatogram raw data in a simple, lightweight binary format. However, the fast access comes at a cost of the initial step of producing the cached data structure and potentially lost portability between different processor architectures. We therefore recommend to produce the binary format as intermediate format only and not for long-term data storage. The processing time to produce the cached file is not included in our measurements and needs to be considered in real-world applications as well (in general this time is similar to the time the “indexed” or the “event-driven” API takes to process a single file).

### Event-driven processing

Since not all algorithms need random access to mass spectrometric data, we have implemented an API for sequential (event-driven) parsing of mzML and mzXML files. Just as SAX (Simple API for XML) parsers allow client code to handle a specific XML tag as soon as it is encountered, our API allows client code to handle spectra and chromatograms as soon as they are read from disk. This allows for very efficient processing of mzML or mzXML files even in cases where a plain mzML (without an index) or a compressed file (where the index offsets do not match the compressed stream) is encountered. The ability to efficiently process compressed files with minimal memory overhead makes this implementation suitable for high throughput algorithms or for applications in constrained memory and/or disk space environments.

One potential application of the event-driven approach is to chain multiple processing steps directly to the read event. Each individual processing step corresponds to a C++ or Python class implementing a specific operation on a spectrum (or chromatogram). The processing functions of these classes will then be called sequentially, therefore implementing whole data analysis pipelines through this approach. We implemented one example for such a pipeline by chaining (i) a smoothing operation (ii) a peak picking operation and (iii) output to disk, allowing us to retrieve a spectrum immediately after it has been read in, process it and write out the processed result to disk while only one single spectrum is kept in memory at any given time (see S1 Text).

### Improved parsing speed of mzML/mzXML

**Fig 1:**
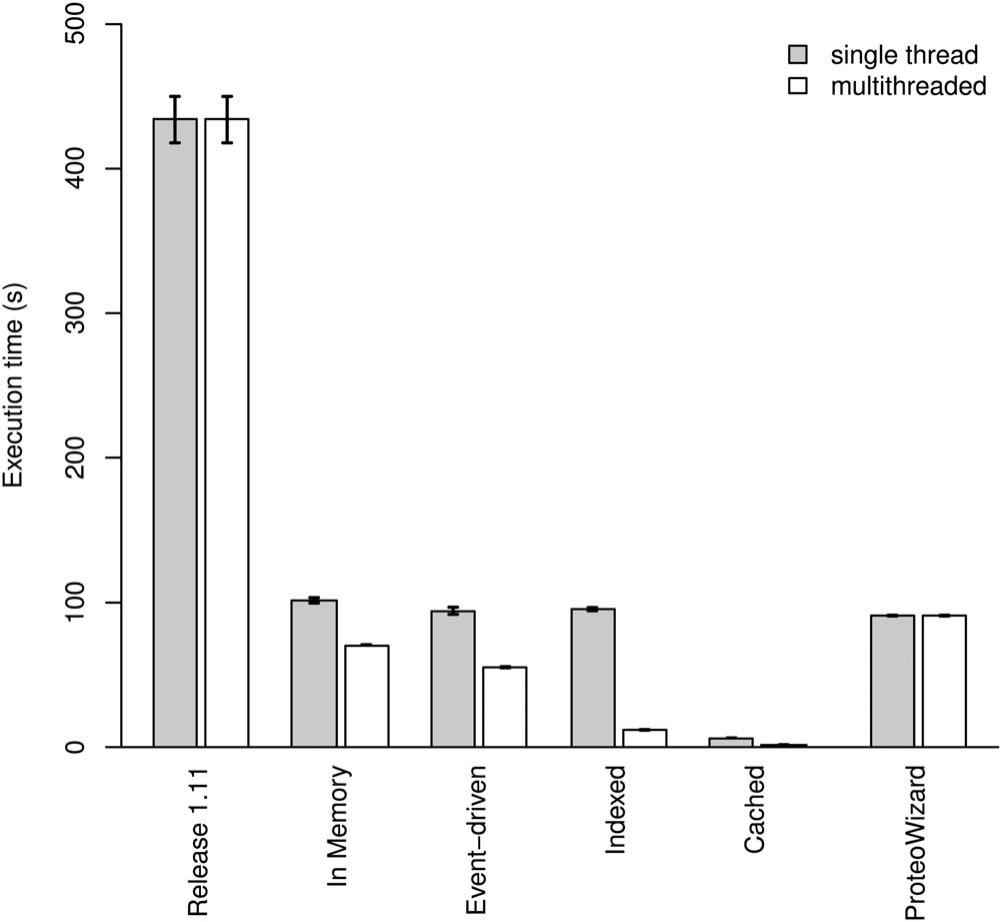
Processing time (s) for the different algorithms. The time to process a single 6.9 GB file (containing 456 million peaks) is depicted for the different algorithms with and without multithreading enabled. The processing time using the ProteoWizard library is added for comparison. While our in-memory and our event-driven data access routines are substantially faster than data access using the OpenMS 1.11 code, the novel “cached” data format is an order of magnitude faster than all other data access methods. Note that some implementations, such as OpenMS 1.11 or ProteoWizard, are not capable of utilizing multiple threads. Comparisons were performed using the TICCalculator and files described in the text using a single thread or 16 threads for the multithreaded bar.

**Fig 2:**
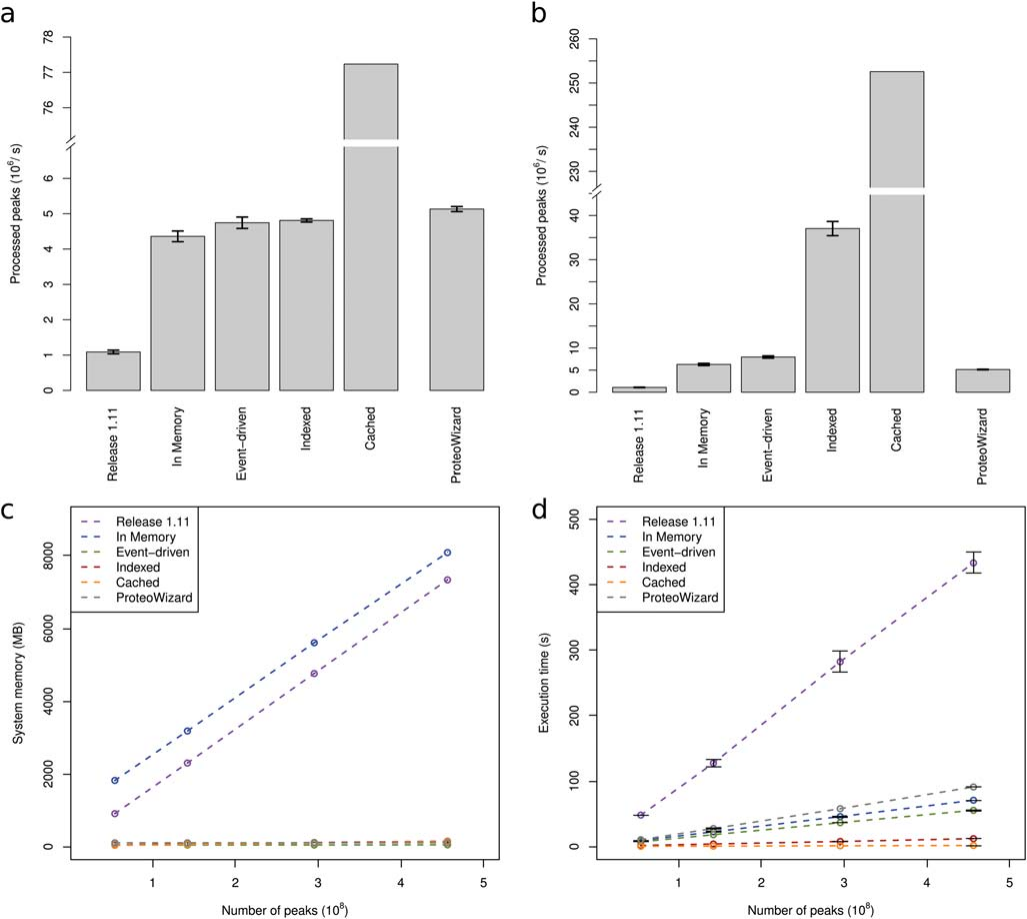
Execution times and memory requirements to calculate the total ion current (TIC) for the described data access implementations. (a)-(b) Processing time normalized to the number of peaks processed per second for the different different implementations using a single thread (a) or up to 16 threads (b). No error bars for “Cached” are depicted due to graphical reasons (see S1 Text). For comparison purposes, the previous implementation (“Release 1.11”) and the ProteoWizard library also depicted (both only support single-threaded XML parsing). The best performing implementation (“Cached”) provides a speed-up of more than 200-fold when using multiple threads. (c)-(d) Memory requirements (c) and execution times (d) as a function of the number of peaks for the different algorithms. While runtime scales linearly for all algorithms, the “event-driven” and “cached” algorithms have constant memory requirements. The implementations were allowed to use parallel processing (up to 16 threads). Comparisons were performed using the TICCalculator and files described in the text.

During our work on the OpenMS core, we also implemented several substantial performance optimizations that increase the processing speed of the mzXML and mzML parsing modules in OpenMS. We have documented these instances and produced patches for the upstream project, thus making our improvements in parsing available for all future releases of OpenMS (Figs 1 and 2, see comparison to “OpenMS 1.11”). These improvements of the OpenMS code allow it to decode XML data in parallel when multiple computing cores are available. Furthermore, we identified several performance bottlenecks which we have eliminated through optimized string parsing, identification and speed-up of “hot-paths” through the code as well as the omission of unnecessary checks that decreased performance.

### Application

To show the versatility and applicability of our improvements, we have implemented an algorithm that reports summary statistics about a mass spectrometric run such as the number of peaks measured or the total ion current (TIC). We chose a question that required iterating through every single peak in a file and therefore would allow straight-forward comparison in CPU time and memory usage between the four different implementations for raw data access. The benchmark results (Fig 1) show that our novel API can achieve speedups of up to 200-fold in read speed while consuming constant memory; specifically our “cached” implementation was able to completely process a 7 GB mzML file in 1.3 seconds using less than 15 MB of memory (Fig 2). Even a complete SWATH-MS file from Röst et al. [14] with a file size of 60 GB (containing 3.95 billion peaks) was processed in 15 seconds using less than 20 MB of system memory with the cached algorithm (compared to 92 seconds using the “indexed” algorithm and to 8 minutes using the event-driven algorithm; the “in-memory” algorithm could not be compared due to system memory limits).

Next, we benchmarked our improved OpenMS kernel against the widely used ProteoWizard software library [15, 16] by implementing an algorithm that uses the ProteoWizard API to read the raw data and then computes the TIC on the given input file. In a single-threaded environment, all implementations (improved OpenMS and ProteoWizard, except “cached”) showed almost equal processing performance (maximal performance difference was 15%). Notably, the “cached” implementation proved to at least a factor of 15 faster than all other implementations (see Fig 1). Additionally, when using multi-threaded parsing, the improved OpenMS kernel is substantially faster than the ProteoWizard kernel (Fig 2a and 2b). The measured speed-up compared to ProteoWizard ranged from 20% to 50% (“in-memory” and “event-driven”) to 7 fold and almost 50 fold (“indexed” and “cached”).

Our results show a clear improvement of our modified OpenMS kernel compared to the 1.11 kernel. Depending on the chosen access mode, a speedup of 200-fold was achieved and a reduction from *O*(*n*) memory complexity to constant *O*(1) memory complexity was obtained for specific implementations (Fig 2). Compared to the memory requirement of over 7.9 GB, all three newly implemented access methods had much lower requirements with the “cached” method at 15 MB, the “event-driven” processing at 49 MB and the “indexed” mzML implementation at 150 MB. In terms of processing speed, all three new implementations and the original “in-memory” implementation were faster than the OpenMS 1.11 kernel. Speedups ranged from over 200 fold (“caching”) to 4 fold for the “in-memory” parsing.

## Discussion

In conclusion, we present a set of major improvements to the XML parsing module of OpenMS, an important, community-driven open-source project in computational mass spectrometry. Specifically, we address the challenges of processing datasets produced by next-generation mass spectrometry instruments such as SWATH-MS enabled instruments, allowing the improved OpenMS kernel to process data produced by these instruments with ease. We chose OpenMS as a framework since it provides a mature codebase in two different programming languages (C++ and Python) and any improvement not only benefits future developers in both languages but can already impact existing tools using the OpenMS framework. Furthermore, the speed of our improved XML parser compares favorably to that of other projects, such as ProteoWizard [15, 16] or pymzML [25] which both also implement random access to raw data (see Fig 2 as well as S1 Text).

The proposed APIs provide real benefits in processing speed and memory requirements, essentially transforming linearly growing memory requirements to constant ones and at the same time improving parsing speed more than 4-fold compared to the previous state-of-the-art. With the proposed software, developers in Python and C++ can now choose between four different models of accessing raw mass spectrometric data, each tailored to the needs of specific algorithms or environments. We provide an access mode that is optimized for sequential data processing (“event-driven”), one access mode optimized for frequent random access (“cache”), one access mode for infrequent random access (“indexed”) and the original implementation suited for small-scale data (“in-memory”), see Table 1. All implementations (except the original one) excel by requiring only limited memory and allow processing of datasets that are much larger than system memory. We therefore expect that current and future algorithmic implementations in computational proteomics can directly profit from our flexible and scalable mass spectrometric XML parsing library.

## Supporting Information

S1 Text **Supplemental Code examples.** Code examples in C++ and Python providing minimal examples on how to use the described API.
**Supplemental Discussion.** Further discussion containing a comparison between the C++ and Python implementations, considerations regarding random access reads in large files and a comparison to other libraries parsing mass spectrometric XML files.
**Supplemental Figures.** Further figures comparing the OpenMS API to the ProteoWizard API and comparing the Python API to the C++ API.
(PDF)

S1 Code Benchmark program to measure the performance of the Python implementation. (ZIP)
