# Supplementary material for: Fast and Efficient XML Data Access for Next-Generation Mass Spectrometry
Source: PLoS One. 2015 Apr 30;10(4):e0125108. doi: 10.1371/journal.pone.0125108 (PMC4416046; doi:10.1371/journal.pone.0125108)
Supplement: S1 Text — Supplemental Code examples. Code examples in C++ and Python providing minimal examples on how to use the described API. Supplemental Discussion. Further discussion containing a comparison between the C++ and Python implementations, considerations regarding random access reads in large files and a comparison to other libraries parsing mass spectrometric XML files. Supplemental Figures. Further figures comparing the OpenMS API to the ProteoWizard API and comparing the Python API to the C++ API. (PDF) [file pone.0125108.s001.pdf]

Fast and Efficient XML Data Access for Next-Generation Mass Spectrometry

Hannes L. Röst,[1,2] Uwe Schmitt,[3] Ruedi Aebersold,[1,4,5] and Lars Malmström[1,6,*]

[1]*Department of Biology, Institute of Molecular Systems Biology, ETH Zurich, CH-8093 Zurich, Switzerland*

[2]*Ph.D. Program in Systems Biology, University of Zurich and ETH Zurich, CH-8057 Zurich, Switzerland*

[3]*ID Scientific IT Services, ETH Zurich, CH-8092 Zurich, Switzerland*

[4]*Competence Center for Systems Physiology and Metabolic Diseases, CH-8093 Zurich, Switzerland*

[5]*Faculty of Science, University of Zurich, CH-8057 Zurich, Switzerland*

[6]*S3IT, University of Zurich, CH-8057 Zurich, Switzerland*

[*]Corresponding author: lars.malmstroem@s3it.uzh.ch; Phone: +41 44 635 4252

Contents

I. Code availability and examples 2
A. C++ code 3
B. Python code 10

II. Supplemental Discussion 14
A. Comparison between C++ and Python libraries 14
B. Considerations regarding random access reads in large files 14
C. Comparison to other libraries 16

References 17

III. Supplemental Figures 17

I. CODE AVAILABILITY AND EXAMPLES

All code described in this manuscript is available at `https://github.com/OpenMS/OpenMS`. A sample application as well as all code implemented in the manuscript to perform benchmarking can be downloaded from `https://github.com/hroest/OpenMS/tree/feature/measureMemoryConsumption` (for the sample Application, see `https://github.com/hroest/OpenMS/blob/feature/measureMemoryConsumption/src/utils/ExampleApp.cpp`).

For the algorithm used to produce the performance values for ProteoWizard, please see `https://github.com/hroest/pwiz_readspeed`. For the code used to compare the Python implementations, see the supplementary `TICCalculator.py` file.

A. C++ code

See Listing 1 for example C++ code used to calculate the TIC using the in-memory API.

See Listing 2 for example C++ code used to calculate the TIC using the indexed mzML API.

See Listing 3 for example C++ code used to calculate the TIC using the indexed mzML API and multiple threads for faster execution.

See Listing 4 for example C++ code used to calculate the TIC using the cached API.

See Listing 5 for example C++ code used to calculate the TIC using the "event-based" streaming API.

Listing 1 TIC calculation using in-memory access. The following code describes our C++ implementation in OpenMS which calculates the TIC using the "in memory" algorithm.

```
String in = "input.mzML";
MzMLFile mzml;
MSExperiment<> map;
// load data from a regular MzML file
mzml.load(in, map);
double TIC = 0.0;
long int nr_peaks = 0;
for (int i =0; i < map.size(); i++)
{
  nr_peaks += map[i].size();
  for (int j = 0; j < map[i].size(); j++)
  {
    TIC += map[i][j].getIntensity();
  }
}
```

Listing 2 TIC calculation using indexed access. The following code describes our C++ implementation in OpenMS which calculates the TIC using the "random access" algorithm using an indexed mzML file.

```
String in = "input.mzML";
IndexedMzMLFileLoader imzml;
// load data from an indexed MzML file
OnDiscMSExperiment<> map;
imzml.load(in, map);
double TIC = 0.0;
long int nr_peaks = 0;
for (int i =0; i < map.getNrSpectra(); i++)
{
  OpenMS::Interfaces::SpectrumPtr sptr = map.getSpectrumById(i);
  nr_peaks += sptr->getIntensityArray()->data.size();
  TIC += std::accumulate(sptr->getIntensityArray()->data.begin(),
                         sptr->getIntensityArray()->data.end(), 0.0);
}
```

Listing 3 TIC calculation using parallel indexed access. The following code describes our C++ implementation in OpenMS which calculates the TIC using the "random access" algorithm using an indexed mzML file and makes use of parallelization.

```
    String in = "input.mzML";
    IndexedMzMLFileLoader imzml;
    // load data from an indexed MzML file
    OnDiscMSExperiment<> map;
    imzml.load(in, map);
    double TIC = 0.0;
    long int nr_peaks = 0;
    // create parallel loop (each thread has its own ‘‘map’’ variable)
#pragma omp parallel for firstprivate(map)
    for (int i =0; i < map.getNrSpectra(); i++)
    {
      OpenMS::Interfaces::SpectrumPtr sptr = map.getSpectrumById(i);
      // store TIC and number of peaks in local variables
      double nr_peaks_local = sptr->getIntensityArray()->data.size();
      double TIC_local = std::accumulate(sptr->getIntensityArray()->data.begin(),
                                         sptr->getIntensityArray()->data.end(), 0.0);

      // block all threads and add to global counter
#pragma omp critical (indexed)
      {
        TIC += TIC_local;
        nr_peaks += nr_peaks_local;
      }
    }
```

Listing 4 TIC calculation using cached indexed access. The following code describes our C++ implementation in OpenMS which calculates the TIC using the "random access" algorithm using a cached mzML file.

```
String in = "input.mzML";
CachedmzML cache;
std::ifstream ifs(in.c_str(), std::ios::binary);

// read the cached file and retrieve indices
cache.createMemdumpIndex(in);
const std::vector<std::streampos> spectra_index = cache.getSpectraIndex();

double TIC = 0.0;
long int nr_peaks = 0;
for (int i=0; i < spectra_index.size(); i++) {
  BinaryDataArrayPtr mz_array(new BinaryDataArray);
  BinaryDataArrayPtr intensity_array(new BinaryDataArray);
  int ms_level = -1;
  double rt = -1.0;
  // move the file pointer to the correct location
  ifs.seekg(spectra_index[i]);
  // get the raw data from the cached file
  CachedmzML::readSpectrumFast(mz_array, intensity_array, ifs, ms_level, rt);
  nr_peaks += intensity_array->data.size();
  TIC += std::accumulate(intensity_array->data.begin(), intensity_array->data.end(), 0.0);
}
```

Listing 5 TIC calculation using sequential (streaming) access. The following code describes our C++ implementation in OpenMS which calculates the TIC using the "event-based" streaming algorithm. Note that the objects described in Listing 6 needs to be available for this code to work.

```
String in = "input.mzML";
TICConsumer consumer;
MzMLFile mzml;

// Perform the work
mzml.transform(in, &consumer);

// Read out the result
double TIC = consumer.TIC;
long int nr_peaks = consumer.nr_peaks;
```

Listing 6 Code necessary to implement the streaming data acces. The following code describes our C++ implementation in OpenMS which calculates the TIC using the "event-based" streaming algorithm.

```
class TICConsumer :
    public Interfaces::IMSDataConsumer< MSExperiment<> > {
public:
  double TIC;
  long int nr_peaks;
  // Create new consumer, set TIC and number of peaks to zero
  TICConsumer() :
    TIC(0.0),
    nr_peaks(0.0) {}

  // For each spectrum, add the total intensity to the TIC variable
  void consumeSpectrum(MSExperiment<>::SpectrumType & s) {
    for (int i = 0; i < s.size(); i++) {
      TIC += s[i].getIntensity();
    }
    nr_peaks += s.size();
  }

  void consumeChromatogram(MSExperiment<>::ChromatogramType&) {}
  void setExpectedSize(size_t expectedSpectra, size_t expectedChromatograms) {}
  void setExperimentalSettings(const ExperimentalSettings& exp) {}
};
```

B. Python code

See Listing 7 for example Python code used to calculate the TIC using the in-memory API.

See Listing 8 for example Python code used to calculate the TIC using the indexed mzML API.

See Listing 9 for example Python code used to calculate the TIC using the cached API.

See Listing 10 for example Python code used to calculate the TIC using the "event-based" streaming API.

Listing 7 TIC calculation using Python in-memory access. The following code describes our implementation in OpenMS which calculates the TIC using the Python "in memory" algorithm.

```
import pyopenms
exp = pyopenms.MSExperiment()
pyopenms.MzMLFile().load("infile.mzML", exp)
TIC = 0.0
nr_peaks = 0
for spec in exp:
    TIC += sum(spec.get_peaks()[1])
    nr_peaks += spec.size()
```

Listing 8 TIC calculation using Python indexed access. The following code describes our implementation in OpenMS which calculates the TIC using the Python "random access" algorithm using an indexed mzML file.

```
import pyopenms
exp = pyopenms.OnDiscMSExperiment()
pyopenms.IndexedMzMLFileLoader().load("infile.mzML", exp)
TIC = 0.0
nr_peaks = 0
for i in range(exp.getNrSpectra()):
    spec = exp.getSpectrum(i)
    TIC += sum(spec.get_peaks()[1])
    nr_peaks += spec.size()
```

Listing 9 TIC calculation using cached indexed access in Python. The following code describes our implementation in OpenMS which calculates the TIC using the Python "random access" algorithm using a cached mzML file.

```
import pyopenms
exp = pyopenms.SpectrumAccessOpenMSCached("infile.mzML")
TIC = 0.0
nr_peaks = 0
for i in range(exp.getNrSpectra()):
    spec = exp.getSpectrumById(i)
    TIC += sum(spec.getIntensityArray())
    nr_peaks += len(spec.getIntensityArray())
```

Listing 10 TIC calculation using the Python interface The following Python code calculates the TIC using the "event-based" streaming algorithm.

```python
class TICCalculator:

    def __init__(self):
        self.TIC = 0
        self.nr_peaks = 0

    def consumeSpectrum(self, s):
        self.TIC += sum(s.get_peaks()[1])
        self.nr_peaks += s.size()

    def consumeChromatogram(self, s):
        pass

    def setExpectedSize(self, x, y):
        pass

    def setExperimentalSettings(self, e):
        pass

functor = TICCalculator()
pyopenms.MzMLFile().transform("infile.mzML", functor)
TIC = functor.TIC
nr_peaks = functor.nr_peaks
```

II. SUPPLEMENTAL DISCUSSION

A. Comparison between C++ and Python libraries

In Figure 4 we compare the performance of the different APIs provided in OpenMS through pyOpenMS [1] in terms of performance. As expected, processing speed of Python is slightly slower than C++, however the new pyOpenMS execution times are also substantially improved over the 1.11 OpenMS kernel. Only the cached implementation in Python offered a substantial speed gain over C++, but the improvement was not as large as observed for a pure C++ implementation.

B. Considerations regarding random access reads in large files

Some algorithms need random access reads into large raw data files that cannot be easily bundled or ordered by retention time. In these cases, random access to data is necessary which precludes the algorithm from using the in memory implementation (due to system memory restrictions) and the event-driven implementation (since random access is necessary). In these cases, using the indexed data access API – which relies on the `mzML_idx` standard – is the most straight-forward way to implement such an algorithm. The `mzML_idx` standard stores binary offsets to the individual data tags inside the mzML file which allows a file seek to jump to the desired location and read the next XML tag (either a `<spectrum>` or `<chromatogram>` tag).

However, using the `mzML_idx` standard has at least two main disadvantages. (i) The file needs to be in de-compressed form while reading since the indices relate to the de-compressed locations and stream-based compression algorithms such as gzip do not allow random access. (ii) During each read the raw data has to be converted from a base64 string into a floating point number representation in memory which is generally the most time consuming step while reading. If many random access operations need to be performed, these two disadvantages necessitate initial de-compression of the file and then only allow relatively slow access to each spectrum.

Therefore, we implemented the "cached" file format that allows fast caching of the raw data while retaining the meta data structure of mzML. The file format consists of two linked files, a `cachedMzML` which only contains the raw mass spectrometric data and an associated

TABLE I: **Execution time for C++ implementations.** The execution times for different mzML readers implemented in C++ are compared (best of 3) on a file with a size of 800 MB. The dev version refers to the development version of OpenMS described in the main text, or in the case of ProteoWizard to the SVN revision 7261 (committed 2015-03-06).

Software	Version	Access Mode	Execution Time (1 thread)	Execution Time (16 threads)
OpenMS	1.11	In Memory	47.6 s	47.6 s
ProteoWizard	dev (r7261)		10.45 s	10.45 s
OpenMS	dev	In Memory	13.0 s	8.97 s
OpenMS	dev	Indexed	11.18 s	1.55 s
OpenMS	dev	Event-driven	11.98 s	7.0 s
OpenMS	dev	Cached	0.7 s	0.27 s

`mzML` file which does not contain any raw data (only the meta-data is retained in the XML data structure). By allowing for clear separation of raw data and meta-data, reading the meta-data into memory and performing search operations (for example collecting all spectra within a certain retention time window, collecting all spectra with their precursor masses in a certain range etc.) is extremely fast since the data structures are very small (generally a few MB) and no raw data needs to be loaded into memory for this operation. Once a suitable set of spectra (or a single spectrum) is found, its associated raw data can be loaded from the disk from the `cachedMzML` file for further processing. Loading the raw data of specific spectra from disk can be extremely fast as indicated by Figure 1 and 2 in the main text, which indicate that loading the cached raw structures can be more than 10 times faster than any other access mechanism. As we describe in the main text, we were able to process the raw data of all spectra of a 60 GB `mzML` file and compute the TIC on this data in less than 20 seconds using the cached access algorithms.

TABLE II: **Execution time for Python implementations.** The execution times for different mzML readers in Python are compared (best of 3) on a file with a size of 800 MB. All tests were performed on the same RHEL system also used in the main text. The “dev” versions indicate that we used OpenMS with the enhancements described in the main text. Note that the “OpenMS” software relates to the C++ implementation (shown as comparison) while pyOpenMS relates to the Python implementation.

Software	Version	Access Mode	Execution time
pymzML	0.7.4	SAX parsing	35.6 s
pyOpenMS	1.11	In Memory	49.5 s
pyOpenMS	dev	In Memory	14.3 s
pyOpenMS	dev	Indexed	20.4 s
pyOpenMS	dev	Event-driven	18.4 s
pyOpenMS	dev	Cached	5.6 s
OpenMS	1.11	In Memory	47.6 s
OpenMS	dev	In Memory	13.0 s

C. Comparison to other libraries

In order to assess the performance of our implementation, we compared it to the XML parsing implementation available in the ProteoWizard software, another major open-source data access library [2, 3]. We used the ProteoWizard library revision 7261 to build a custom program that calculates the TIC and compared it to the performance measured using the OpenMS implementations. The results of the measurement are shown in the main text, in Figure 1 and in Table I.

Our results indicate that the single threaded execution of ProteoWizard and OpenMS are on par in terms of processing speed (except the “cached” implementation which is an order of magnitude faster). However when using multiple threads, OpenMS is 30 % (“In Memory”), 60 % (“event-driven”) or even a factor of 4 (“indexed”) and 50 (“cached”) faster.

We also compared our implementation to pymzML [4] which only provides a feature-complete mzML reader. Interestingly, when run on the same machine as the other comparisons, we found pymzML to outperform the Python and C++ OpenMS 1.11 implemen-

tations (see Table II). These results are consistent with the ones reported in Bald et al. [4] who found that OpenMS and pymzML perform similarly. However, after applying the modifications reported in this article, we find that the Python implementations based on pyOpenMS are substantially faster than pymzML (see Table II). We observed speedups of two-fold or more (up to six fold for the cached Python implementation). In addition, we conclude that the Python wrappers do not carry a large overhead in performance as the difference between the C++ (OpenMS) and Python (pyOpenMS) implementations were about 10 % for the "In Memory" algorithms. For the other access modes, we observed larger overhead time (less than two-fold for "event-driven" and "indexed"). For these comparisons, the `TICCalculator.py` script was used.

---

[1] H. L. Röst, U. Schmitt, R. Aebersold, and L. Malmström, Proteomics **14**, 74 (2014).

[2] M. C. Chambers, B. Maclean, R. Burke, D. Amodei, D. L. Ruderman, S. Neumann, L. Gatto, B. Fischer, B. Pratt, J. Egertson, et al., Nature Biotechnology **30**, 918 (2012).

[3] D. Kessner, M. Chambers, R. Burke, D. Agus, and P. Mallick, Bioinformatics (Oxford, England) **24**, 2534 (2008).

[4] T. Bald, J. Barth, A. Niehues, M. Specht, M. Hippler, and C. Fufezan, Bioinformatics **28**, 1052 (2012).

III. SUPPLEMENTAL FIGURES

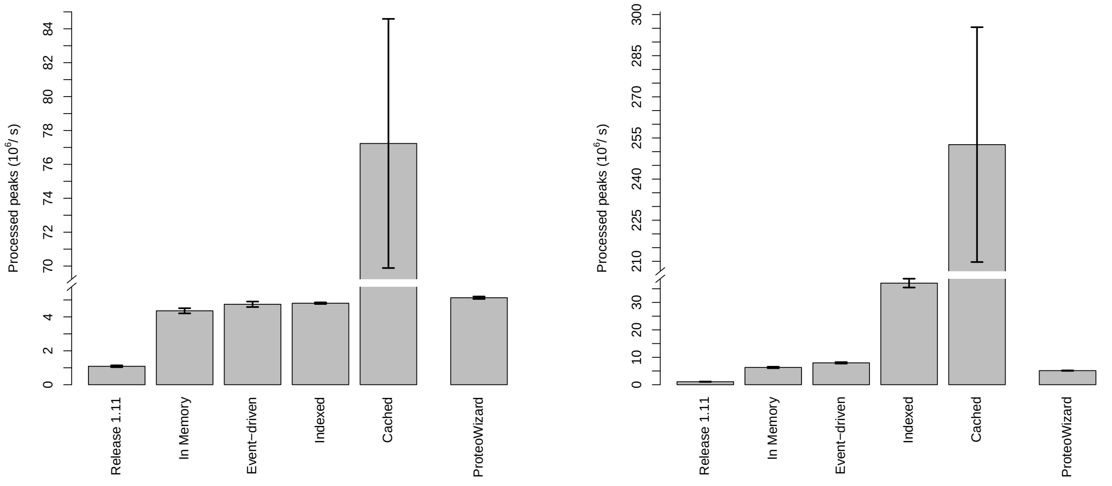

(a) Processing speed (single thread)

(b) Processing speed (multiple threads)

FIG. 1: **Processing speed for the different algorithms.** The processing speed normalized to the number of peaks processed per second for different algorithms (and ProteoWizard for comparison) is depicted. The speed was measured across a range of different mzML files which contained $54.29 \cdot 10^6$ to $456.3 \cdot 10^6$ peaks. For clarity in the display, the figure in the main text does not contain an error bar for the "cached" implementation. Since the "cached" implementation is an order of magnitude faster than the other implementations, additional graphs are provided here with the error bar drawn to scale.

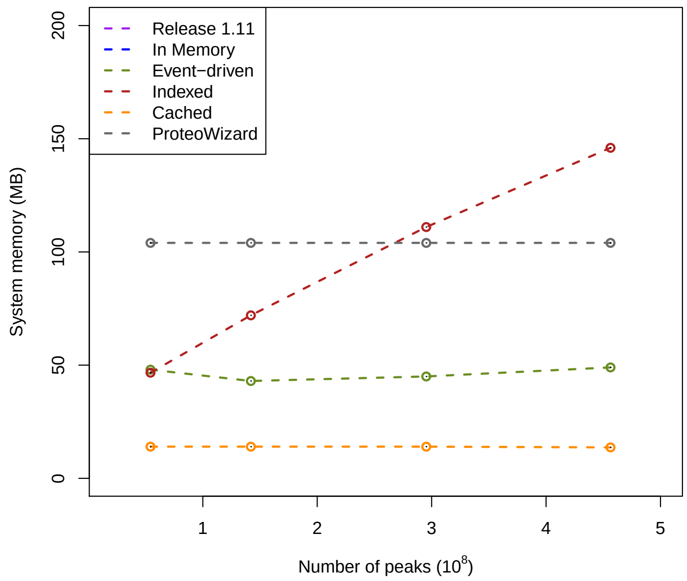

FIG. 2: **Memory requirements for the low-memory algorithms.** Memory requirements as a function of the number of peaks for the low-memory algorithms. The "event-driven" and "cached" implementations show linear behavior in memory, independent of the number of peaks in a file. The "indexed" implementation shows a slight increase in memory consumption with file size since it keeps meta-data (previous data processing, retention time, MS1 level *etc.*) for each spectrum and chromatogram in memory. Note that the memory requirements are at least one order of magnitude lower than the file sizes.

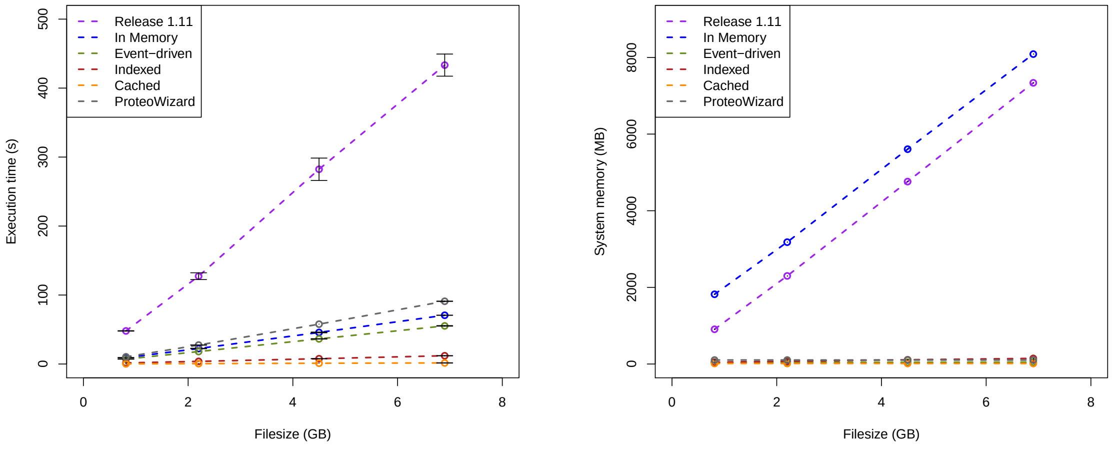

(a) Execution times (walltime) depending on file size

(b) Memory requirements (mega byte) depending on file size

FIG. 3: **Execution times and memory requirements of the described data access implementations.** Execution times and memory requirements as a function of the file size in gigabytes for the different algorithms (instead of number of peaks as in the main text).

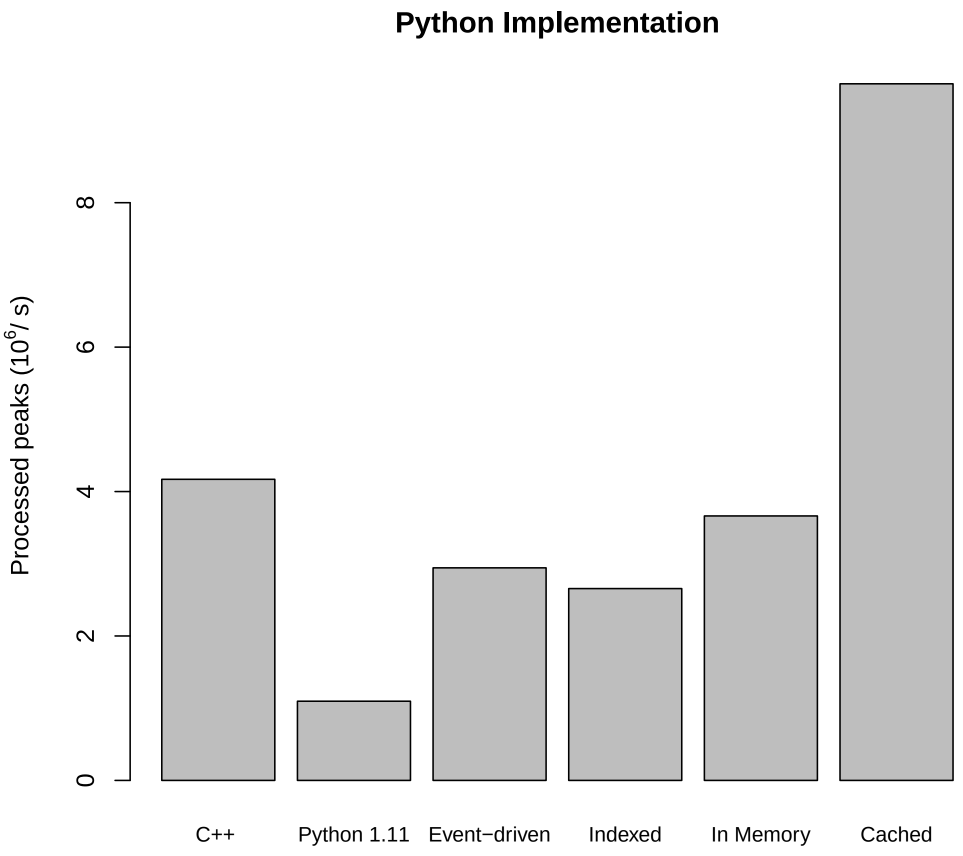

FIG. 4: **Execution times of the Python API.** Execution times of the Python implementations, compared to the C++ implementation in OpenMS. Note how the current processing speed in Python almost matches the C++ processing speed and even exceeds it for the cached implementation. The C++ value in this graph is equivalent to the single threaded value for the "In Memory" algorithm in Figure 1 of the main text.
